# A porcine circovirus type 2d-based virus-like particle subunit vaccine effectively protects pigs against homologous challenge

**DOI:** 10.3389/fmicb.2026.1802883

**Published:** 2026-05-01

**Authors:** Pengfei Chen, Yinghui Sun, Mengya Wang, Chunhua Li, Ye Xia, Huili Liu, Linlin Qi, Fusheng Si, Denian Miao

**Affiliations:** 1 Institute of Animal Science and Veterinary Medicine, Shanghai Academy of Agricultural Science, Shanghai, China; 2 School of Medicine, Shanghai University, Shanghai, China

**Keywords:** porcine circovirus type 2d, virus-like particle, protective immunity, lymphoid depletion, homologous challenge

## Abstract

Porcine circovirus-associated disease (PCVAD), caused by pathogenic porcine circovirus type 2 (PCV2), remains one of the most economically devastating diseases in the global swine industry, and the continued emergence and predominance of the PCV2d genotype have raised concerns regarding the efficacy of currently available vaccine. In this study, a virus-like particle (VLP) subunit vaccine based on the PCV2d capsid protein was developed using a prokaryotic expression system, and its immunogenicity and protective efficacy were systematically evaluated in pigs. Following immunization and subsequent homologous PCV2d challenge, vaccinated pigs exhibited significantly attenuated clinical manifestations, including reduced rectal temperature elevation and less impairment of average daily weight gain during the early phase of infection. Moreover, vaccination resulted in markedly decreased viral DNA loads in serum and multiple tissues, including the lungs, spleen, kidneys, and inguinal and mesenteric lymph nodes, compared with unvaccinated challenged controls. The VLP vaccine also induced robust humoral immune responses, characterized by elevated PCV2-specific IgG and neutralizing antibody titers, and effectively mitigated PCV2d-associated histopathological lesions in the lungs and lymph nodes. Importantly, immunized pigs showed alleviation of peripheral T-lymphocyte depletion, with higher levels of CD3^+^, CD4^+^, and CD8^+^ T cells following challenge. Collectively, these findings demonstrate that the PCV2d capsid-based VLP subunit vaccine confers effective protection against PCV2d infection and represents a promising candidate for the prevention and control of PCVAD.

## 1 Introduction

Porcine circovirus type 2 (PCV2) is the primary etiological agent of porcine circovirus-associated disease (PCVAD), which affects pigs of all ages and manifests as a spectrum of clinical syndromes, including postweaning multisystemic wasting syndrome, porcine dermatitis and nephropathy syndrome, congenital tremor in piglets, respiratory and digestive system diseases in fattening pigs, and reproductive disorders in sows, causing considerable economic losses to the global pig farming industry (Opriessnig et al., 2007; Segales, 2012; de Sousa Moreira et al., 2022; Lim et al., 2022). More importantly, as one of the significant pathogens of porcine viral infectious diseases, PCV2 mainly damages the immune system of pigs, resulting in immune dysfunction or immunosuppression. This immunocompromised state predisposes infected pigs to secondary pathogens such as porcine parvovirus, porcine reproductive and respiratory syndrome virus, and mycoplasma, thereby complicating disease management and posing additional challenges to the prevention and control of pig infectious diseases (Vincent et al., 2003; Rose et al., 2012; Ouyang et al., 2019).

PCV2 belongs to the genus *Circovirus* within the family *Circoviridae*, which is one of the smallest non-enveloped DNA viruses with a diameter of 16–18 nm in an icosahedral form (Tischer et al., 1982; Opriessnig et al., 2020). The genome of PCV2 is a single-stranded circular DNA approximately 1.76 kb in length with a stem-loop structure (Finsterbusch and Mankertz, 2009). Approximately 10 open reading frames (ORFs) have been detected in the ambisense genome, among which ORF1 encodes one or more replication-associated proteins on the viral strand, whereas ORF2 encodes the capsid protein (Cap) on the complementary replicative intermediate DNA strand (Guo et al., 2022). PCV2 has high evolutionary dynamics as a single-stranded RNA virus, and can currently be classified into eight genotypes based on ORF2 sequence diversity. Of these, PCV2a, PCV2b, and PCV2d are the predominant circulating genotypes, while PCV2c, PCV2e, PCV2f, PCV2g, and PCV2h are detected less frequently (Harmon et al., 2015; Davies et al., 2016; Franzo et al., 2016; Franzo and Segales, 2018; Jang et al., 2021). Before 2003, the prevalent genotypes were mainly PCV2a and PCV2b, with PCV2a being the dominant genotype. PCV2b, which has increased virulence, subsequently became the predominant genotype resulting from the first genotypic shift (An et al., 2007; Carman et al., 2008; Constans et al., 2015). From 2010–2015, PCV2 underwent a second genotypic shift, with the main prevalent strain, PCV2b, being gradually replaced by PCV2d and becoming the dominant prevalent genotype worldwide (Xiao et al., 2015; Franzo et al., 2016; Kwon et al., 2017).

Given the widespread prevalence of PCV2 in pig farms, vaccination is regarded as the main effective method to reduce PCV2 prevalence and thereby control PCVAD (Dvorak et al., 2016). In general, commercialized vaccines for PCV2 are classified into two main categories: inactivated vaccines and subunit vaccines, most of which are based on the immuno-genic properties of the Cap protein (Chae, 2012; Guo et al., 2022). Early commercialized vaccines on the international market for PCV2 infection were based on PCV2a genotype, examples including Circovac^®^, the world's first PCV2 vaccine developed by Merial (France); FosteraTM PCV, an inactivated PCV1–2a chimeric vaccine produced by Pfizer Animal Health, Inc, USA; and Ingelvac CircoFLEX, developed by Boehringer Ingelheim. These vaccines have been reported to reduce clinicopathological manifestations and enhance growth performance in pig herds (Fraile et al., 2012; Hemann et al., 2012; Caspari et al., 2014; Seo et al., 2014; Opriessnig et al., 2017). However, following the widespread use of PCV2a-based vaccines, global genotypic shift from PCV2a to PCV2b occurred accompanied by severe clinical symptoms in vaccinated herds, indicating that PCV2a-based vaccines could not provide complete cross-protection against PCV2b (Carman et al., 2008; Opriessnig et al., 2013). In China, the first approved PCV2 vaccine was the PCV2a inactivated vaccine (strain LG) developed by the Harbin Veterinary Research Institute of the Chinese Academy of Agricultural Sciences in 2010 (Guo et al., 2015). Subsequently, several PCV2b inactivated vaccines, based on such strains as SH, DBN-SX07, WH, CP08 and YZ, have been approved and applied (Guo et al., 2022). Under the enormous pressure of persistent PCV2 infection and vaccination, genotypic shifts frequently occur to evade the host immune response (Franzo et al., 2016).

Over the past decade, the prevalence of the PCV2d subtype has increased worldwide. However, vaccines specifically targeting PCV2d remain limited in China and are insufficient to meet current clinical and market demands (Peng et al., 2023). Accumulating evidence indicates that PCV2a- and PCV2b-based vaccines do not confer complete cross-protection against PCV2d infection, whereas PCV2d-based VLP vaccines elicit stronger humoral and cellular immune responses and provide superior protection against homologous challenge (Kim and Hahn, 2021; Kim et al., 2023). Given the ability of the PCV2 Cap protein to self-assemble into VLPs *in vitro*, coupled with their favorable immunogenicity and safety profiles, the development of a cost-effective PCV2d-based subunit vaccine is highly warranted. In the present study, we successfully developed a PCV2d VLP-based subunit vaccine via a prokaryotic expression system. This candidate vaccine effectively alleviated lymphoid depletion and conferred significant protection against PCV2d challenge in pigs, thereby underscoring its considerable potential for future clinical application.

## 2 Materials and methods

### 2.1 Cells, viruses and antibodies

The PCV-free PK-15 cells (ATCC: CCL-33) used in this study were cultured in modified Eagle's medium (MEM, Life Technologies, 11095098) supplemented with 10% fetal bovine serum (FBS, Gibco) at 37°C in a humidified atmosphere of 5% CO_2_. The PCV2d strain JSM (GenBank accession no. PQ114731) used for challenge and neutralization assays was isolated from a swine farm affected by PCVAD. Flow cytometry antibodies, including Fixable Viability Dye eFluor™ 506 (65-0866-14), CD3 monoclonal antibody (FITC, MA541029), CD4 monoclonal antibody (PE, MA528733), and CD8 alpha monoclonal antibody (PE-Cyanine5, MA528716), were purchased from Invitrogen. The mouse anti-PCV2 Cap protein monoclonal antibody used for western blotting was kindly provided by Qingdao Animal Health Protection National Engineering Technology Research Center Company Limited.

### 2.2 Expression and purification of PCV2d-Cap protein

The PCV2d capsid protein was expressed using a prokaryotic expression system. Briefly, the full-length ORF2 gene of the PCV2d HB-MC1 strain (GenBank accession no. KM460824.1) was codon-optimized for expression in Escherichia coli, synthesized, and subcloned into the expression vector pET30a (+) (HG-VYN0173, Novagen) to generate the recombinant plasmid pET30a (+)-Cap2d. The plasmid was then transformed into *E. coli* Rosetta (DE3) competent cells (ZC125-1, ZOMANBIO). Protein expression was induced by adding isopropyl β-D-1-thiogalactopyranoside (IPTG, RT108-01, TIANGEN) to a final concentration of 0.2 mM, followed by incubation at 30°C for 6 h. The bacterial cells were harvested by centrifugation at 8,000 × g for 10 min at 4°C, and the pellet was resuspended in lysis buffer (200 mM NaCl, 50 mM Tris-HCl, 10 mM imidazole, pH 8.0) at a ratio of 1:10 (w/v). The resuspended cells were disrupted by sonication (200–300 W, with 3-s pulses followed by 5-s intervals to prevent overheating) on ice for 20–30 min until the suspension became clear and translucent. The lysate was centrifuged at 10,000 × g for 20 min at 4°C, and the supernatant was collected and filtered through a 0.22 μm filter. After the selective binding of the protein onto a Ni-NTA column (Yeasen, Shanghai, China), elution was achieved by applying a linear gradient of Elution buffer (200 mM NaCl, 50 mM Tris-HCl, 10 mM-500 mM imidazole, pH 8.0). The eluate was concentrated, analyzed by SDS-PAGE, dialyzed to remove imidazole, and stored at −80°C.

### 2.3 Western blotting

The proteins separated by SDS-PAGE in the gel were transferred onto nitrocellulose membrane using an eBlot L1 protein transfer system (GenScript). The membrane was blocked with 5% non-fat milk in Tris-buffered saline-Tween (TBST) and incubated with a monoclonal antibody against the PCV2-Cap at room temperature for 1 h. Then, the membrane was washed using TBST for three times and incubated with horseradish peroxidase (HRP)-conjugated secondary antibody (SA00001-1, Proteintech) for 1 h. The protein signals were detected with enhanced chemiluminescence detection kits (15159, Thermo Scientific) and visualized by the Amersham ECL Western Blotting Analysis System (GE Healthcare, Chicago, IL, USA).

### 2.4 TEM and DLS analysis

The self-assembled PCV2d-based VLPs were identified by transmission electron microscopy (TEM). A small droplet (5–10 μL) of the purified PCV2d-Cap protein solution was placed onto a carbon-coated copper grid and allowed to adhere for 1–2 min. Excess liquid was carefully blotted away with filter paper. The grid is then stained by applying a drop of 2% phosphotungstic acid for about 30–60 s. The excess stain is blotted off, and the grid was air-dried completely. Finally, the prepared grid was loaded into the TEM for observation. Particle diameter and size distribution of PCV2d-based VLPs were also evaluated by dynamic light scattering (DLS) using a Zetasizer Nano ZS instrument (Malvern Instruments Limited, Worcestershire, UK) 25°C. The Z-average is represented as diameter in nm ± width of the distribution.

### 2.5 Immunization and PCV2d challenge in piglets

**Figure 1:**
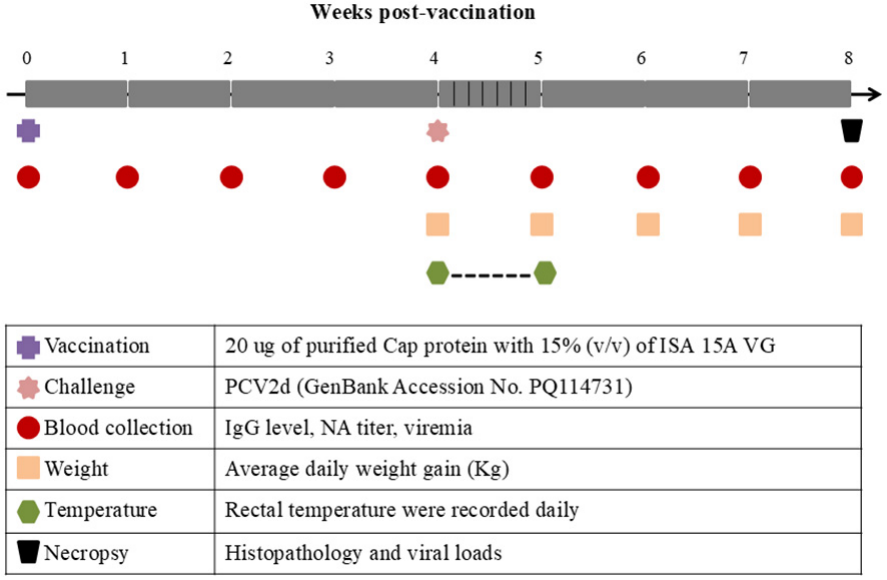
Schematic diagram of the immunization and challenge experimental design. 4-week-old piglets were randomly allocated into three experimental groups: the Vac+PCV2 group, the PCV2 group, and the control group. Three piglets in the Vac+PCV2 group received a single intramuscular vaccination in the neck region. 4 weeks post-vaccination, four piglets in both the Vac+PCV2 and PCV2 groups were challenged intranasally with 2 mL of PCV2d (10^5^ TCID_50_/mL) administered into each nostril. The control group remained unchallenged throughout the study.

The 4-week-old piglets used in this study were purchased from conventional farms that were confirmed to be negative for PRRSV, PCV2, PCV3, PCV4, Mycoplasma spp, and PCV2-specific IgG antibodies. The piglets were randomly divided into three groups, vaccinated and challenged (hereafter called the “Vac+PCV2”, *n* = 3), unvaccinated and challenged (hereafter called the “PCV2”, *n* = 4), and unvaccinated and unchallenged (hereafter called the “Control”, *n* = 4) groups. Piglets in the Vac+PCV2 group were immunized intramuscularly in the neck with a 1 mL dose of PCV2d subunit vaccine containing 20 μg of purified Cap protein with 15% (v/v) of ISA 15A VG adjuvant (Seppic, France). At 4 weeks post-vaccination (WPV), piglets in Vac+PCV2 group and PCV2 group were challenged with PCV2d at a dose of 2 mL per piglet (10^5^ TCID_50_), administered equally into each nostril, while piglets in the control group were mock challenged. Importantly, all pigs were immunized with 1.0 ml of keyhole limpet hemocyanin (0.5 mg/ml) emulsified in Incomplete Freund's Adjuvant at four sites (both armpits and both hips) per animal 3 days before the challenge, as well as 3 and 6 days post-challenge. Peripheral blood was collected before and weekly thereafter. Following viral challenge, the clinical signs and rectal temperature were recorded daily, and body weights were measured weekly (Figure 1). All the surviving pigs were humanely euthanized at 8 weeks post-vaccination and necropsied to evaluate pathological lesions and viral DNA loads in tissues, including lung, spleen, kidney, inguinal and mesenteric lymph nodes.

### 2.6 Enzyme-linked immunosorbent assay (ELISA)

All the serum samples were tested for PCV2 antibodies via a commercial indirect enzyme-linked immunosorbent assay (ELISA) kit (JN60315, Beijing Jinnuobaitai Biotech Company Limited, China) according to the manufacturer's directions. Briefly, all the serum samples were diluted 1:100 in the sample diluent. Subsequently, 100 μL/well of the diluted serum was added to an ELISA plate coated with the purified PCV2 capsid protein, and incubated at 25°C for 30 min. After three washes with the washing solution, 100 μL/well of the horseradish peroxidase-labeled goat anti-pig IgG antibody was added to the ELISA plate and then further incubated at 25°C for 30 min. Following three washing steps with the washing solution, the color reaction was developed with 100 μL/well of the tetramethyl benzidine solution at 25°C for 15 min. Finally, 50 μL/well of the stop solution was added to the ELISA plate, and the absorbance was read at 450 nm via a microplate reader (51119080, Thermo Scientific).

### 2.7 PCV2 neutralizing antibody detection

An indirect immunofluorescence assay was performed to determine the viral neutralizing antibody titers of the serum samples collected in the present study. Briefly, all the serum samples were inactivated by heating at 56°C for 30 min, followed by 2-fold serial dilutions from 1:2^1^ to 1:2^15^. One hundred microlitres of viral stock solution (200 TCID_50_) was mixed with an equal volume of diluted serum in a 96-well plate and incubated at 37°C for 1 h. After incubation, the virus-serum mixture was added to the 70–80% confluent monolayer of PK-15 cells at 37°C for 72 h with 5% CO_2_. PK-15 cells were washed with phosphate-buffered saline (PBS) for three times and fixed with cold methanol at 4°C for 30 min. Then, the plates were incubated with fluorescein isothiocyanate (FITC)-conjugated PCV-2 polyclonal antiserum (VMRD, USA) at 37°C for 1 h. After being washed with PBS, the plates were observed using an Evos fluorescence microscope (M7000; Thermo Fisher Scientific, Waltham, MA, USA). Neutralizing antibody titers were determined as the reciprocal of the highest serum dilution that blocks 50% viral infection.

### 2.8 Flow cytometry analysis

Blood was collected from the anterior vena cava of each pig using anticoagulant-treated tubes. Subsequently, 5 mL of Red Blood Cell Lysis Buffer was added to lyse red blood cells on ice for 5 mins to isolate peripheral blood mononuclear cells (PBMCs). For flow cytometry analysis, 1 million PBMCs were stained with Fixable Viability Dye eFluor™ 506 (eBioscience, Invitrogen) to discriminate dead cells, along with fluorescently labeled monoclonal antibodies-anti-CD3 (FITC, Invitrogen), anti-CD4 (PE, Invitrogen), and anti-CD8 (PE-Cyanine5, Invitrogen)-in PBS containing 0.5% BSA on ice for 30 min. After incubation, cells were washed twice with FACS buffer. Data acquisition and analysis were performed using a CytoFlex flow cytometer (Beckman).

### 2.9 Quantification of PCV2 DNA

Viral DNA from peripheral blood and different tissues including lung, spleen, kidney, inguinal and mesenteric lymph nodes was extracted via a commercial FastPure Viral DNA/RNA Mini Kit (Vazyme) according to the manufacturer's protocol. Quantitative polymerase chain reaction (qPCR) assays were performed using TB Green^®^ Premix Ex Taq™ (TaKaRa) with a LightCycler 480 system (Roche Diagnostics) to analyze viremia and viral loads. Briefly, a PCV2d ORF1 fragment was cloned into the pET-30a vector to construct a standard plasmid. The plasmid concentration was initially quantified and adjusted to 1 × 10^10^ copies/μL, followed by preparation of serial 10-fold dilutions to serve as DNA templates for standard curve generation. The standard curve was established by plotting the cycle threshold (Ct) values obtained from amplifying these template dilutions against the logarithm of their corresponding copy numbers. Finally, PCV2 genome copy numbers in unknown samples were determined by interpolating their Ct values onto this standard curve. The ORF1-specific forward primer 5′-CCGCAGTATTCTGATTAC-3′ and reverse primer 5′-TGGAAATTCAGGGCATG-3′ were used, and the reaction program was performed according to the following conditions: predenaturation at 95°C for 30 s, followed by denaturation at 95°C for 5 s and annealing at 60°C for 20 s for up to 40 cycles.

### 2.10 Histological and Immunohistochemical Staining

All pigs were humanely euthanized at 28 days post-challenge (DPC) for pathological examinations. Tissues, including lung, inguinal and mesenteric lymph nodes, from each pig were collected and routinely fixed in 10% formalin for 36 h at room temperature. Then the paraffin-embedded tissues were prepared as described previously. After the paraffin-embedded tissues were cut, deparaffinized, rehydrated, and routinely stained with hematoxylin and eosin, the sections were scanned for analysis. After the paraffin-embedded tissues were cut, deparaffinized, rehydrated, and routinely stained with hematoxylin and eosin, the sections were scanned for analysis. Sections (5 μm) of formalin-fixed paraffin-embedded tissues were blocked with 1% BSA for 1 h at room temperature, and then incubated with mouse anti-PCV2 Cap monoclonal antibody overnight at 4°C, followed by incubation with peroxidase-labeled goat anti-mouse IgG secondary antibody in a humidified chamber for 1 h at room temperature. Finally, the samples were visualized with a 3, 30-diaminobenzidine (DAB) chromogen kit, and counterstain was performed with hematoxylin.

### 2.11 Statistical analysis

All statistical analyses were performed using GraphPad Prism 7 (GraphPad Software, La Jolla, CA, USA). Statistical significances were analyzed using one-way or two-way ANOVA. Data are expressed as the mean ± standard error of the mean (SEM). Differences were considered statistically significant at *P* < 0.05.

## 3 Results

### 3.1 Production and characterization of PCV2d-based VLPs

**Figure 2:**
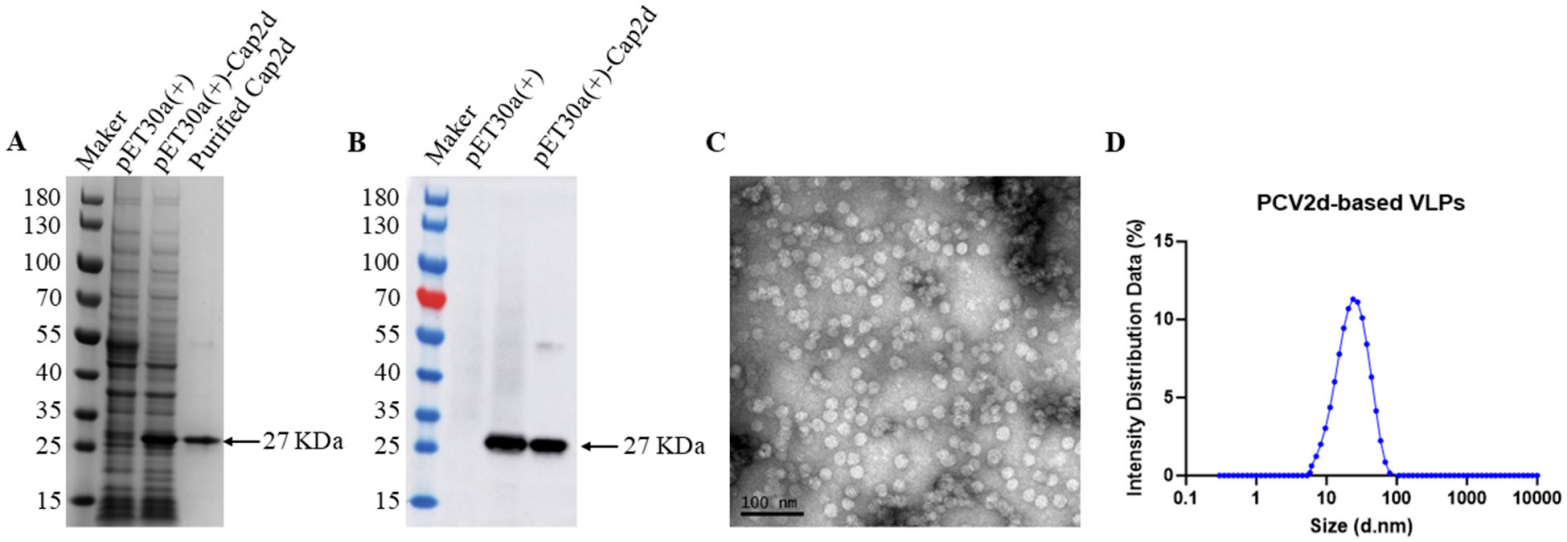
Preparation and characterization of PCV2d-based VLPs. The solubility and purity of the purified PCV2d capsid (Cap) protein were evaluated through sodium dodecyl sulfate-polyacrylamide gel electrophoresis (SDS-PAGE) analysis **(A)**. The integrity and specificity of the purified Cap protein were further confirmed by Western blotting using a monoclonal antibody against the PCV2 capsid antigen **(B)**. The self-assembled VLPs formed by PCV2d-Cap proteins were morphologically characterized via transmission electron microscopy (TEM) **(C)**. Particle diameter and size distribution of PCV2d-based VLPs were analyzed by dynamic light scattering (DLS) **(D)**.

Following induction and purification, the obtained PCV2d-Cap proteins showed soluble and high purity, as demonstrated by SDS-PAGE (Figure 2A). Western blotting analysis using a specific monoclonal antibody against the PCV2-Cap protein confirmed the identity of the protein, indicating that the molecular weight of the monomeric full-length PCV2d-Cap protein was approximately 27 KDa as expected (Figure 2B). PCV2d-Cap proteins expressed in E. coli can self-assemble to form VLPs, as shown by transmission electron microscopy (Figure 2C). DLS revealed a homogenous population of non-aggregated particles with an average diameter of 23.97 nm for PCV2d-based VLPs (Figure 2D), which is consistent with the TEM results.

### 3.2 Clinical evaluation of rectal temperature and average daily weight gain

**Figure 3:**
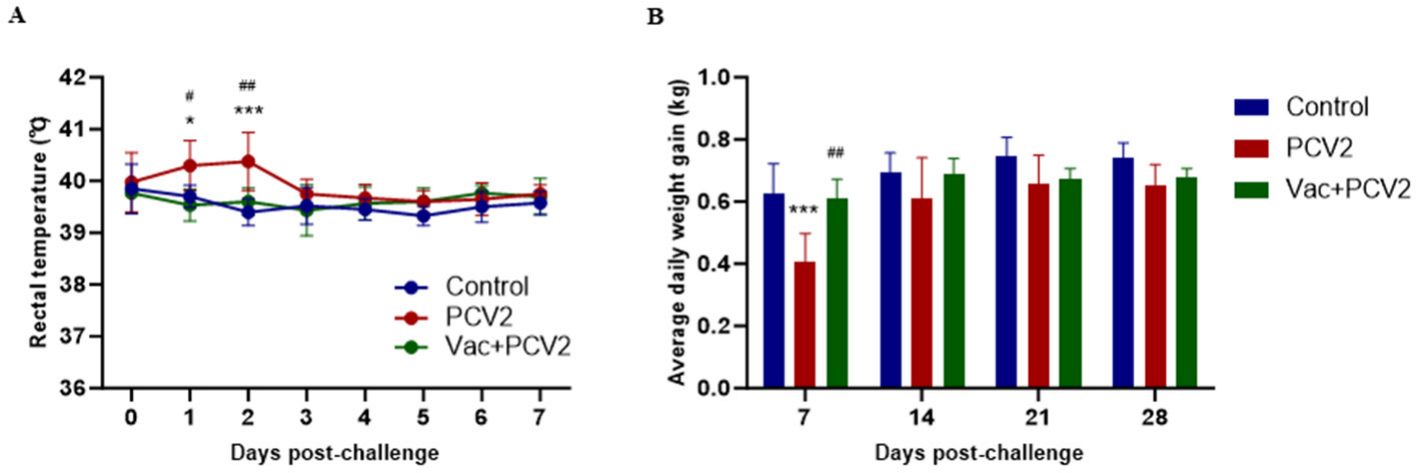
Rectal temperature and average daily weight gain of piglets after challenge. Changes in the average rectal temperature of piglets in each group within the first 7 DPCs **(A)**. Changes in the average daily weight gain of the piglets in each group are shown every 7 days over 28 days **(B)**. The error bars represent standard deviations, and differences were analyzed using two-way ANOVA. The statistical differences between PCV2 and Control are indicated by * (*, *P* < 0.05; ***, *P* < 0.001). The statistical differences between PCV2 and Vac+PCV2 are indicated by ^#^ (^#^, *P* < 0.05; ^##^, *P* < 0.01).

After challenge, all pigs in the Vac+PCV2 group maintained a normal body temperature similar to those in the control group, and no notable clinical signs, including depression, anorexia, epilepsy, vomiting or diarrhea, were observed throughout the study. However, pigs in the PCV2 group presented a high fever during the first two DPC, as the rectal temperature of some piglets exceeded 40.5°C as shown in Figure 3A. The body temperature of pigs in the PCV2 group subsequently returned to normal, with no significant difference from that of the control group. The weight of each pig was measured every week post-challenge, and the results revealed no significant difference in the average daily weight gain between the Vac+PCV2 group and the control group. However, growth was significantly retarded in the PCV2 group compared with the Vac+PCV2 group and the control group, especially in the first week post-challenge (Figure 3B).

### 3.3 Measurement of PCV2-specific antibodies and neutralizing antibodies

**Figure 4:**
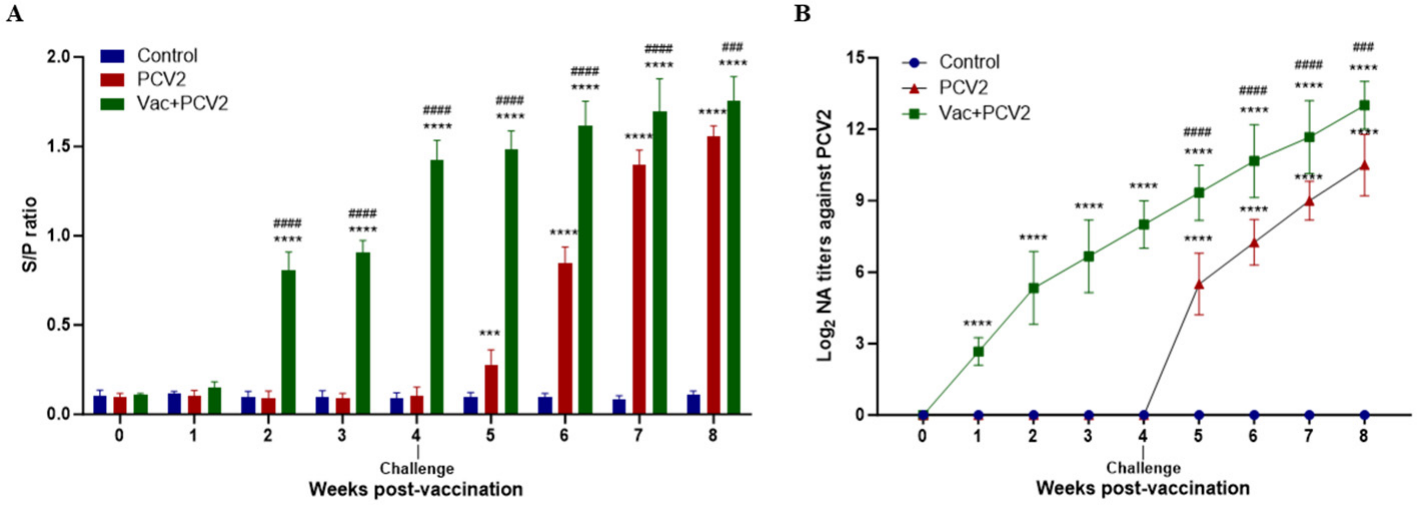
Serum IgG antibody levels and viral neutralizing antibody titers at different weeks post-vaccination. PCV2-specific antibodies in serum samples collected weekly after vaccination were measured via a commercial ELISA kit **(A)**. Viral neutralizing antibody titers in serum samples collected weekly after vaccination were analyzed by indirect immunofluorescence assay **(B)**. The error bars represent standard deviations, and differences were analyzed using two-way ANOVA. The statistical differences between PCV2 and Control, as well as between Vac+PCV2 and Control, are indicated by * (***, *P* < 0.001; ****, *P* < 0.0001), and the statistical differences between PCV2 and Vac+PCV2 are indicated by ^#^ (^###^, *P* < 0.001; ^####^, *P* < 0.0001).

Before vaccination, no anti-PCV2 antibodies were detected in any of the pigs in the three groups. In the Vac+PCV2 group, seroconversion to PCV2-specific IgG antibodies was observed at 2 WPV, followed by a rapid increase at 4 WPV after which the level remained high. In the PCV2 group, PCV2-specific IgG antibodies were first detected at 5 WPV, and increased substantially at 7 and 8 WPVs. Nevertheless, the PCV2-specific IgG levels in the PCV2 group were significantly lower than those in the Vac+PCV2 group, and the control group remained seronegative throughout the experimental period (Figure 4A). At the time of challenge, pigs in the Vac+PCV2 group presented high neutralizing antibody titers, which continued to increase during the challenge period. In the PCV2 group, PCV2-specific neutralizing antibody titers were first detected at 5 WPV at high levels and continued to increase up to 8 WPV. However, their neutralizing antibody titers were substantially lower than those of the Vac+PCV2 group (Figure 4B). No neutralizing antibodies were detected in the control group throughout the study.

### 3.4 Detection of viremia and tissue viral loads

**Figure 5:**
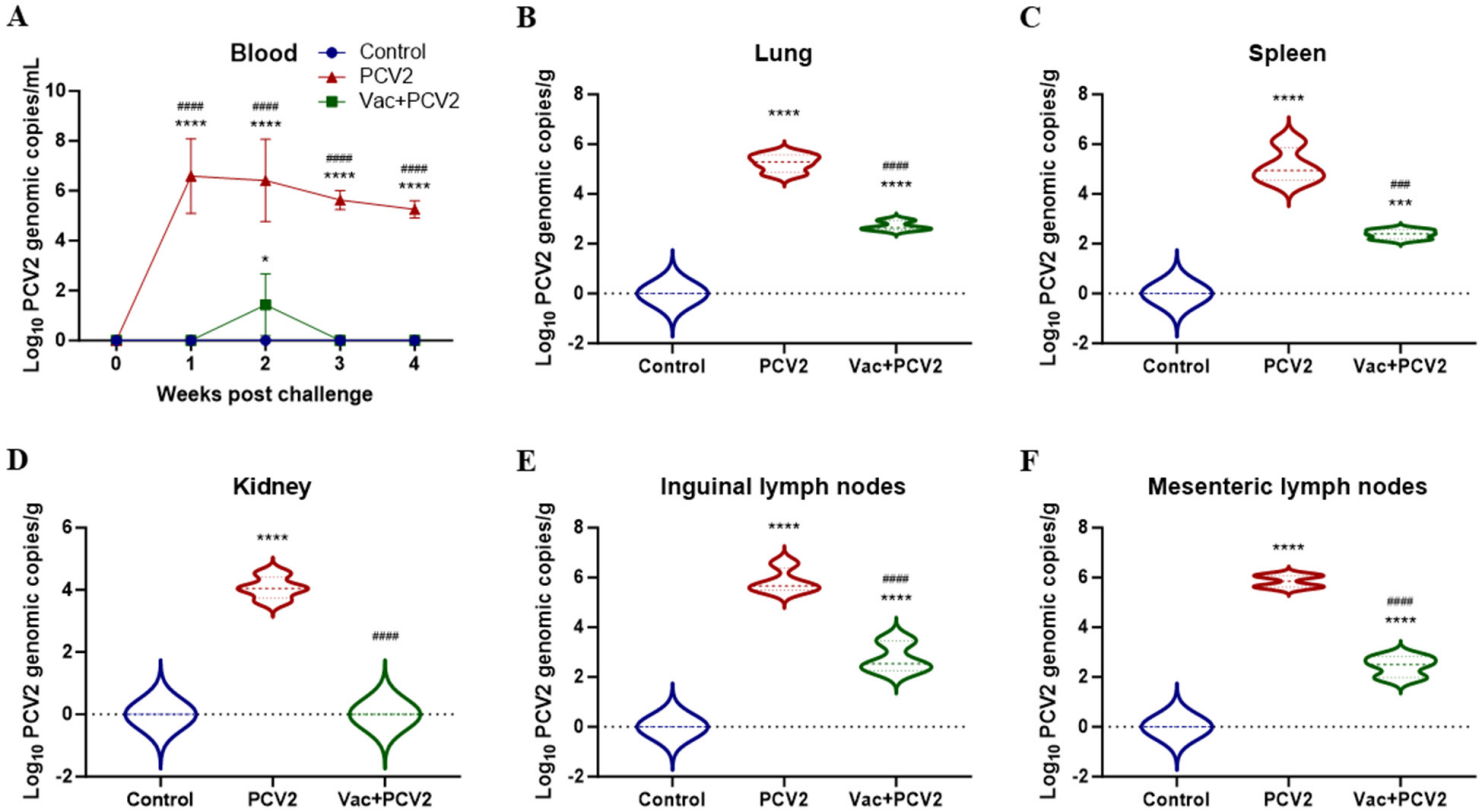
Viremia and viral loads of PCV2 in different tissues. PCV2 genome copies in peripheral blood samples collected from each group at different WPCs were analyzed by qPCR **(A)**. Mean values of the PCV2 genomic copy number in the lung **(B)**, spleen **(C)**, kidney **(D)**, inguinal **(E)** and mesenteric lymph nodes **(F)** at different weeks post-vaccination. The error bars represent standard deviations, and differences were analyzed using two-way ANOVA **(A)** or one-way ANOVA **(B-F)** The statistical differences between PCV2 and Control, as well as between Vac+PCV2 and Control, are indicated by * (*, *P* < 0.05; ***, *P* < 0.001; ****, *P* < 0.0001), and the statistical differences between PCV2 and Vac+PCV2 are indicated by ^#^ (^###^, *P* < 0.001; ^####^, *P* < 0.0001).

Quantitative PCR was performed to quantify PCV2 genome copies in peripheral blood and tissue samples. As shown in Figure 5A, before the challenge, no PCV2 genomes were detected in peripheral blood samples from any of the groups. After challenge, pigs in the PCV2 group presented significantly increased levels of PCV2 DNA, ranging from 1.78 × 10^5^ copies/mL to 3.90 × 10^6^ copies/mL, and peaked at 1 week post-challenge (WPC), whereas PCV2 DNA copies were almost undetectable in pigs in the Vac+PCV2 group, except for the significantly lower levels of PCV2 genomes at 2 WPC. The viral loads of PCV2 in different tissues, including the lung, spleen, kidney, inguinal and mesenteric lymph nodes, were also evaluated. The PCV2 group presented high numbers of PCV2 genomes in the lung (1.41 × 10^5^ copies/g), spleen (1.35 × 10^5^ copies/g), kidney (1.02 × 10^4^ copies/g), inguinal lymph nodes (5.89 × 10^5^ copies/g) and mesenteric lymph nodes (1.23 × 10^6^ copies/g) (Figures 5B–F, respectively), among which the inguinal and mesenteric lymph nodes presented higher levels of PCV2 DNA than did the kidney. In contrast, the lung, spleen, inguinal and mesenteric lymph nodes of the Vac+PCV2 group presented significantly lower levels of PCV2 DNA copies (ranging from 3.14 × 10^2^ copies/mL to 4.34 × 10^2^ copies/mL) than did those of the PCV2 group, even no PCV2 genomes were detected in the kidney. No PCV2 genomes were detected in peripheral blood samples or different tissues in the control group throughout the study.

### 3.5 Histopathological evaluations of the lungs and lymph nodes

**Figure 6:**
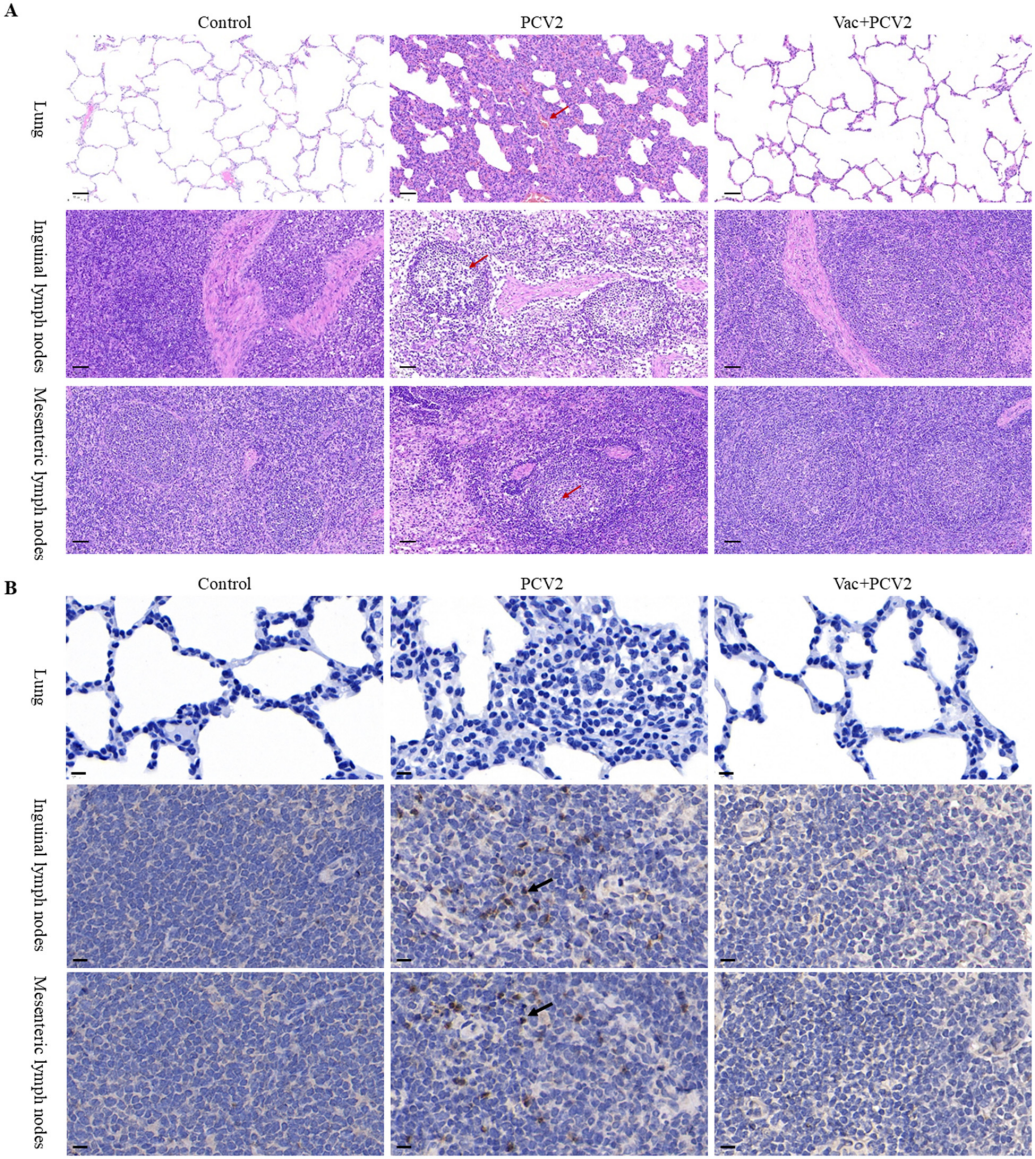
Histopathological evaluation and detection of PCV2 antigen in the lungs and lymph nodes at 4 WPC. Lung tissue as well as inguinal and mesenteric lymph nodes from all experimental groups were subjected to hematoxylin and eosin staining **(A)** and IHC **(B)**. In the PCV2-infected group, prominent interstitial pneumonia and marked lymphoid depletion were observed in the lungs and lymph nodes, respectively (indicated by red arrows). PCV2 antigens were shown by black arrows. Scale bars indicate 50 μm **(A)** and 10 μm **(B)**.

Histopathological examinations of the lungs and lymph nodes of all groups were conducted to systematically and intuitively evaluate the protective effects of PCV2d VLP vaccines against PCV2d challenge. As shown in Figure 6A, lungs in the PCV2 group exhibited typical interstitial pneumonia characterized by alveolar interstitial thickening, peribronchiolar fibroplasia, pulmonary consolidation and infiltration of numerous inflammatory cells in alveolar spaces. In addition, severe lymphoid depletion in inguinal and mesenteric lymph nodes was observed in the PCV2 group. In contrast, no obvious PCV2-associated histopathological lesions were observed in either the Vac+PCV2 group or the control group, indicating effective protection conferred by vaccination.

In the PCV2 group, specific PCV2 antigen signals-visualized as brown cytoplasmic staining-were clearly observed in both the inguinal and mesenteric lymph nodes, indicating active viral replication in lymphoid tissues (Figure 6B). However, no PCV2 antigen was detected in the lung tissue of this group. In contrast, no PCV2 antigen was detected in any of the tissues examined from the Vac+PCV2 group or the control group, demonstrating that vaccination effectively prevented viral antigen distribution in these tissues. These findings suggest that while PCV2 exhibits a tropism for lymphoid tissues following challenge, the PCV2d-based subunit vaccine confers complete protection by preventing viral antigen deposition in these target organs.

### 3.6 Analysis of peripheral blood T lymphocytes subsets

**Figure 7:**
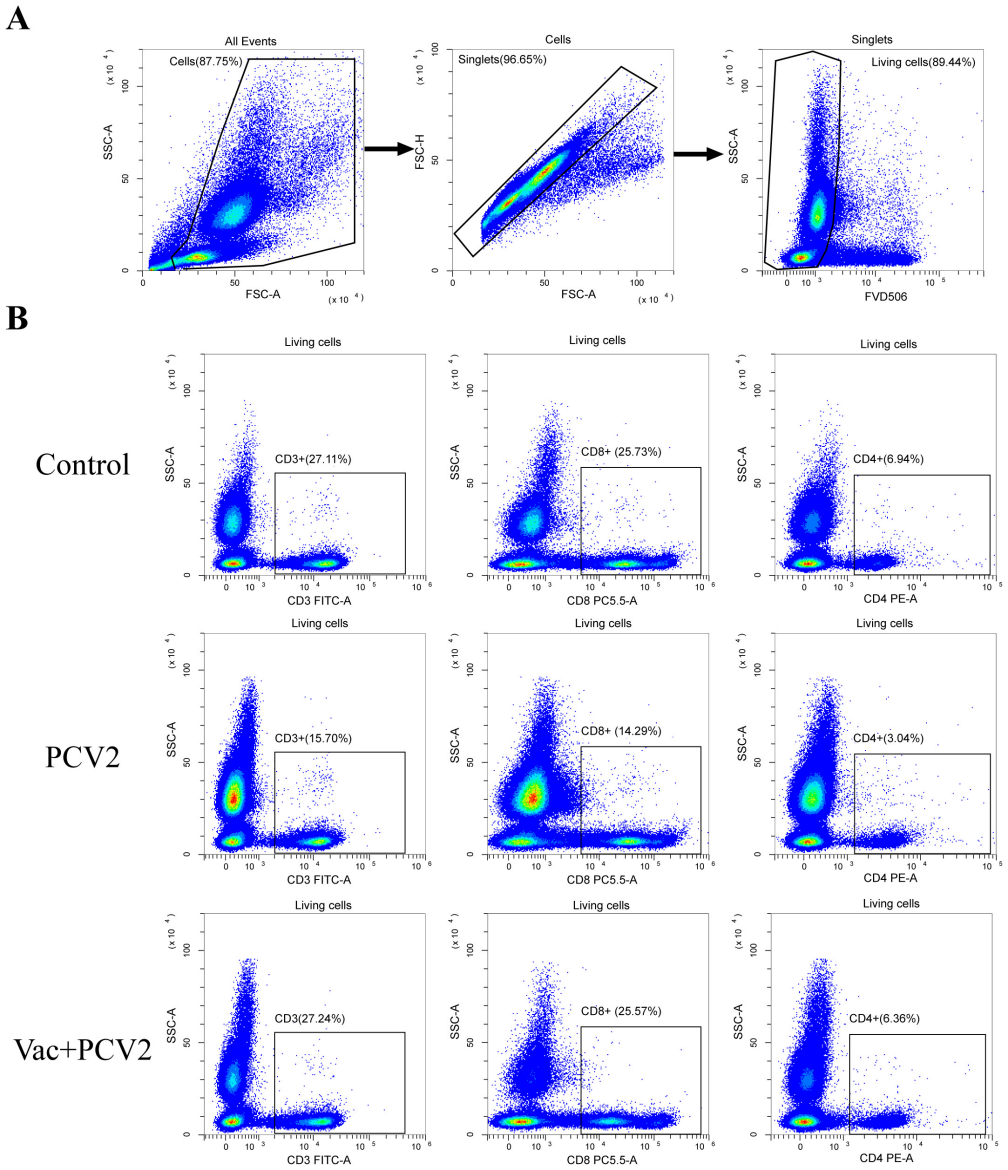
Flow cytometric analysis of peripheral blood T lymphocyte subsets at 1 WPC. Peripheral blood mononuclear cells (PBMC) from pigs in each experimental group were isolated and analyzed to determine the proportions of CD3^+^, CD4^+^ and CD8^+^ T cells. Representative gating strategies and quantitative results are shown. Gating the cell populations to remove cell debris; then gating the single cells to remove adhesive cells; and finally gating the viable Fixable Viability Dye eFluor^TM^ 506-negative cells to remove dead cells **(A)**. Living cells were stained simultaneously with fluorochrome-conjugated monoclonal antibodies against porcine CD3 (FITC), CD4 (PE), and CD8 (PE-Cyanine5), and analyzed by flow cytometry **(B)**.

**Figure 8:**
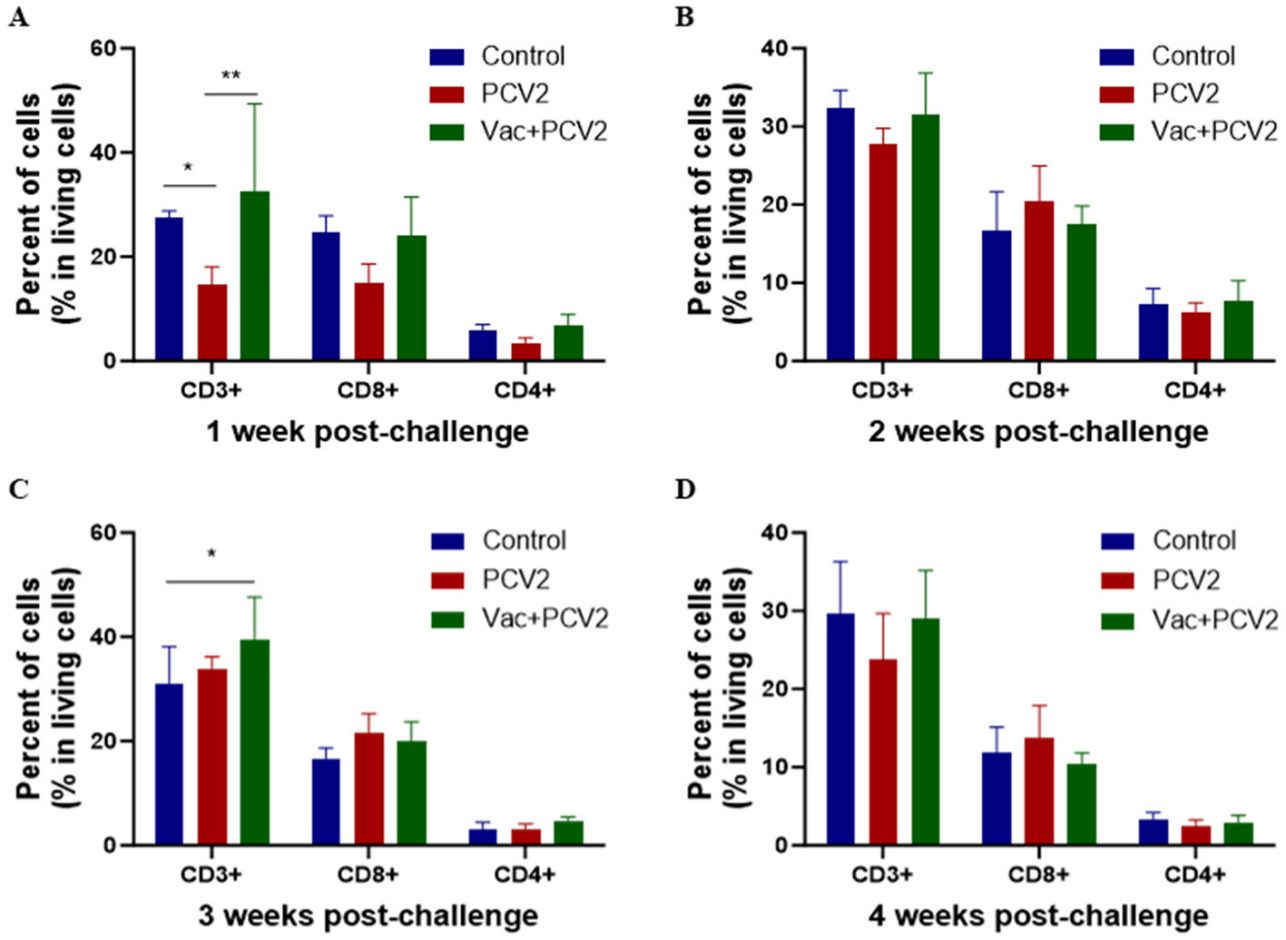
Alterations in the proportions of T lymphocyte subsets among live cells following PCV2 infection at different time points post-challenge. The proportions of T lymphocyte subsets were assessed at **(A)** 1 WPC, **(B)** 2 WPC, **(C)** 3 WPC, and **(D)** 4 WPC. Error bars represent standard deviations. Statistical comparisons among the Control, PCV2, and Vac+PCV2 groups were performed using two-way ANOVA. Significant differences are indicated by asterisks (*P < 0.05; **P < 0.01).

Peripheral blood T lymphocyte subsets, including CD3^+^, CD4^+^ and CD8^+^ T cells, were analyzed and quantified weekly by flow cytometry following PCV2d challenge. At 1 WPC, the proportions of CD3^+^, CD4^+^ and CD8^+^ T cells in the PCV2 group were significantly reduced compared with those in the control group. In contrast, no significant depletion of these T lymphocyte subsets was observed in the Vac+PCV2 group, with levels comparable to those of the control group (Figure 7 and Figure 8A). Over time, the proportion of CD3^+^ T cells in the PCV2 group decreased significantly compared with both the control group and the Vac+PCV2 group at 2 WPC. In contrast, no significant differences in the proportion of CD4^+^ and CD8^+^ T cells were observed among the three groups at this time point (Figure 8B). At 3 and 4 WPC, no significant differences in any T lymphocyte subsets were observed among the groups; however, the proportions of CD3^+^ and CD4^+^ T cells in the PCV2 group remained lower than those in the Vac+PCV2 and control groups at 4 WPC (Figure 8C–D).

## 4 Discussion

The accelerated genetic diversity of PCV2 results from continuous genotype shifts, contributing to the complicated and confusing epidemic status, which poses new challenges for the effective prevention and control of PCV2-associated PCVAD (Franzo et al., 2016; Franzo and Segales, 2018; Almeida et al., 2025). Since 2010, the novel PCV2d has become the predominant genotype in the pig population worldwide as well as in China (Wang et al., 2025). However, most of the vaccines approved on the international market have been developed on the basis of PCV2a or PCV2b genotypes, which have difficulty meeting the actual needs of clinical practice (Kang et al., 2021; Guo et al., 2022; Zheng et al., 2025). Therefore, PCV2d VLP-based subunit vaccines with greater protective efficiency and lower costs are essential. In this study, we found that prokaryotically expressed PCV2d VLPs induced high levels of PCV2-specific antibodies and neutralizing antibodies, and that this subunit vaccine was effective in alleviating clinical symptoms, reducing viremia, viral loads and histopathological lesions, and preventing T lymphocyte depletion in pigs infected with PCV2d. These findings indication against homologous PCV2d challenge.

Neutralizing antibodies induced by vaccination play crucial roles in inhibiting PCV2 replication, alleviating clinical symptoms, reducing viral loads and preventing lymphoid depletion, thereby contributing substantially to protecting immunity against PCV2 infection (Chae, 2012; Park et al., 2019; Kim et al., 2023). A previous study reported that PCV2a-based vaccination can induce neutralizing antibody against multiple PCV2 genotypes, including PCV2a, PCV2b and PCV2d. However, neutralizing antibody titers against PCV2a were consistently higher than those against PCV2b or PCV2d at 21 and 28 DPC (Park et al., 2019). The different neutralizing antibody responses may be attributed to different neutralizing epitopes among PCV2 capsid proteins (Huang et al., 2011; Liu et al., 2013). Sequence analysis have revealed approximately 10% amino acid divergence between PCV2a and PV2d, while PCV2d differs from PCV2b by approximately 7% at both the nucleotide and amino acid (Ju et al., 2023). The antigenic differences resulting from amino acid sequence variations may change the three-dimensional conformation of the cap protein, which ultimately affects the neutralization efficiency of different PCV2 genotypes. Therefore, compared with PCV2a and 2b, PCV2d-based subunit vaccines are more effective at protecting against the currently prevalent PCV2d strains. In the present study, pigs vaccinated with PCV2d-based VLPs elicited a sufficient immune response in terms of PCV2d neutralizing antibody titers, which were significantly greater than those elicited by challenge only throughout the study. In addition, compared with the infection group, the vaccinated group presented much lower levels of PCV2d DNA in the blood, lung spleen, kidney, inguinal and mesenteric lymph nodes, as well as fewer histopathological lesions and alleviated lymphoid depletion of T lymphocytes. These data demonstrate that sufficient neutralizing antibodies produced by vaccination contribute to effective protection against PCV2d infection. Nevertheless, antigenic differences among PCV2d genotype isolates have been observed in neutralization experiments (Ju et al., 2023), highlighting the need to develop a universal vaccine targeting multiple genotypes in the future.

PCV2, the primary causative agent of PCVAD, exerts its pathogenicity primarily through severe immunosuppression, which is induced primarily by targeting and disrupting cells of the monocyte-macrophage lineage and the lymphoid system. Cells of the monocyte-macrophage lineage, including porcine monocytes and alveolar macrophages, are key cellular targets of PCV2 infection. Although these cells support viral persistence and serve as reservoirs, their antigen-presenting and phagocytic functions are often impaired (Meng, 2013). More critically, PCV2 can directly infect and replicate within lymphocytes, which can destroy the architecture of lymphoid follicles, leading to profound lymphoid depletion followed by histiocytic replacement in lymphoid tissues (Hung et al., 2017; Marruchella et al., 2017). Current evidence demonstrates that lymphoid depletion is associated with a reduction in the numbers of multiple immune cells including dendritic cells, natural killer cells, B lymphocytes and T lymphocytes along with an increase in monocytes and granulocytes (Grierson et al., 2007; Darwich and Mateu, 2012). This widespread immunocyte depletion may predispose the host to immunosuppression, leading to further complications in the form of coinfections or secondary infections with bacterial and viral pathogens (Ouyang et al., 2019). In the present study, significant lymphoid depletion was observed in the inguinal and mesenteric lymph nodes as well as in T lymphocyte subsets, including CD3^+^, CD4^+^ and CD8^+^ T cells in the peripheral blood of PCV2-infected pigs. The severity of lymphoid depletion is positively correlated with the amount of PCV2 antigen detected in affected tissues (Meng, 2013), which might further explain why pigs in the PCV2 group presented more severe histopathological lesions and higher levels of viremia than those in the Vac+PCV2 group did.

The depletion and functional impairment of T lymphocytes represent a hallmark of pathogenic PCV2 infection and underpin the severe immunosuppression observed in PCVD (Segales, 2012; Meng, 2013). CD4^+^ T cells are central coordinators of adaptive immune responses and can be classified into several key subsets on the basis of their cytokine secretion profiles and effector functions. Among these, T helper 1 (Th1) cells are characterized by the production of interferon-gamma and tumor necrosis factor-alpha to activate cell-mediated immunity, whereas Th2 cells, characterized primarily by interleukin-4 secretion, promote hu-moral immunity by assisting B cells activation and antibody class switching. Th17 cells, which primarily secrete interleukin-17, play a pivotal role in recruiting neutrophils, while regulatory T (Treg) cells, a specialized CD4^+^ subset often identified by the ex-pression of the transcription factor FoxP3, specialize in suppressing immune responses and maintaining immune homeostasis (Dawson et al., 2013; Yu et al., 2023). A reduction in their quantity and changes in their different subsets severely weaken the coordination ability between humoral immunity and cellular immunity. Recent studies have demonstrated that PCV2 infection promotes the Treg cells differentiation (Cecere et al., 2012; Sinkora et al., 2014), with TGF-β playing a pivotal regulatory role in this process (Liu et al., 2022; Han et al., 2023). Mechanistically, TGF-β has been shown to recruit and phosphorylate Smad3, which subsequently translocates to the nucleus and facilitates Foxp3 transcription, thereby governing Treg cell differentiation (Jiang et al., 2025). In the present study, PCV2-infected piglets presented significant early-stage reductions in T lymphocyte subsets, including CD3^+^, CD4^+^ and CD8^+^ T cells. In contrast, piglets immunized with PCV2d VLP vaccine did not display significant T lymphocyte depletion following PCV2 challenge, suggesting that vaccination effectively preserves T-cell homeostasis during infection. These findings indicate that future research should focus on elucidating the spatiotemporal mechanisms by which vaccination alleviates T lymphocytes depletion during the dynamic infection of PCV2 *in vivo*. A comprehensive understanding of these mechanisms is fundamental to the rational development of effective PCV2 vaccines, which likely confer protection primarily by preventing virus-induced immune dysregulation, preserving T-cell competence, and ensuring robust and coordinated immune protection.

To the end, it should be noted that the vaccinated-and-challenged (Vac+PCV2) group ultimately included three animals, whereas the PCV2 challenge group included four animals. Although this represents the minimum sample size commonly used in large-animal studies, such group sizes are widely considered acceptable in controlled porcine infection models due to practical and ethical constraints. Importantly, consistent protective effects of the vaccine were observed across multiple independent outcome measures. Therefore, while acknowledging the limitation of sample size, the data presented in this study remain sufficient to support the conclusions regarding the protective efficacy of the PCV2d VLP vaccine.

## Data Availability

The original contributions presented in the study are included in the article/supplementary material, further inquiries can be directed to the corresponding author/s.
